# Site-specific occurrence of nonmelanoma skin cancers in patients with cutaneous melanoma

**DOI:** 10.1038/sj.bjc.6602745

**Published:** 2005-08-16

**Authors:** R E Neale, D Forman, M F G Murphy, D C Whiteman

**Affiliations:** 1 Childhood Cancer Research Group, 57 Woodstock Rd, Oxford OX2 6HJ, UK; 2 Centre for Epidemiology and Biostatistics, University of Leeds & Northern and Yorkshire Cancer Registry and Information Service, Arthington House, Hospital Lane, Leeds LS16 6QB, UK; 3 Division of Population Studies and Human Genetics, Queensland Institute of Medical Research, Post Office, Royal Brisbane Hospital, QLD 4029, Australia

**Keywords:** cutaneous melanoma, basal cell carcinoma, skin neoplasms, squamous cell carcinoma, nonmelanoma skin cancer, keratinocytic cancer

## Abstract

In a registry-based case–control study, we compared the site-specific occurrence of nonmelanoma (keratinocytic) skin cancers among patients with cutaneous melanoma cases (cases, *n*=3774) and solid tumours (controls, *n*=349 923), respectively. Overall, patients with melanoma were almost five-fold more likely to develop keratinocytic cancers compared with solid tumour controls (adjusted OR 4.7, 95% CI 4.1–5.3), but the risks varied depending upon the site of melanoma. Whereas patients with melanoma of the head and neck had similarly increased risks of keratinocytic cancers across all body sites, patients with melanoma of the trunk were significantly more likely to develop keratinocyte cancer diagnosed on the trunk (adjusted OR 12.5, 95% CI 7.2–20.2) than on the head and neck (adjusted OR 3.0, 95% CI 2.2–4.3). Similar colocalisation of skin tumours was observed for patients with melanomas of the lower limb. These findings provide support for the hypothesis that skin cancers at different anatomical sites may arise through different causal pathways.

While there is consensus that sunlight is the principal environmental cause of melanomas, their anatomical distribution precludes a simple association with sunlight. Some sites (e.g. trunk) typically have modest levels of sun exposure yet have high melanoma incidence, whereas other highly exposed sites (e.g. hand) have lower melanoma incidence (Green *et al*, 1993; Bulliard *et al*, 1997).

One explanation for these observations may be that the causes of melanoma vary depending upon the anatomical site of the melanocyte The ‘divergent pathway’ model proposes that the relative contribution of genetic and environmental exposures differs according to the anatomic location of the melanoma (Whiteman *et al*, 2003).

The association between sunlight and basal cell carcinoma (BCC) is similarly complex with many lesions occurring on less sun exposed sites, a plateau of incidence with age and a strong association between recreational exposure and risk (Kricker *et al*, 1995). In contrast to melanoma and BCC, the risk of squamous cell carcinoma (SCC) rises monotonically with exposure to the sun.

Patients with melanoma are known to be at a substantially increased risk of the nonmelanoma skin cancers (NMSC) BCC and SCC (Wassberg *et al*, 1996; Levi *et al*, 1997a; Hemminki *et al*, 2003; Crocetti and Carli, 2004) and the converse is also true (Levi *et al*, 1997b, 1998; Wassberg *et al*, 1999; Bower *et al*, 2000; Maitra *et al*, 2005). However, it is not known whether the association between melanoma and NMSC varies according to the anatomical site. We chose to explore this question using a record-linkage study from a British cancer registry. We hypothesised that because of shared risk factors, patients with melanoma of the head or neck would be at particularly high risk of nonmelanoma skin cancer and specifically SCCs.

## MATERIALS AND METHODS

We conducted a registry-based case–control study to test the hypothesis that the risk of site-specific NMSC among people with melanoma varies according to the site of the melanoma. People with melanoma (cases) were compared with people with a solid tumour other than melanoma (controls) for the prevalence of NMSC.

This data-linkage study was conducted using records obtained from the Northern & Yorkshire Cancer Registry and Information Service (NYCRIS). Northern & Yorkshire Cancer Registry and Information Service is responsible for the registration of all malignant cancers in the former Northern and Yorkshire NHS regions and covers a population of 6.4 million. This study was approved by the NYCRIS Advisory Group.

### Case selection

Eligible cases were all notifications of invasive melanoma (ICD10 C43) among people aged 15 years and older diagnosed between January 1986 and December 2000 (99% of diagnoses were histologically confirmed and melanoma registration is estimated to be at least 88% complete (Stefoski Mikeljevic *et al*, 2003). We excluded all cases registered with more than one cancer, apart from NMSC. For the primary analysis, we restricted cases to patients with primary invasive melanoma of the superficial spreading or nodular histological subtypes. We therefore excluded patients diagnosed with metastatic melanomas, lentigo maligna melanomas and rare histological subtypes of melanoma that have not been associated with sun exposure (e.g. acral lentiginous melanoma, desmoplastic melanoma, spindle cell melanoma, etc.) and those without a histological subtype.

### Control selection

Controls were all people diagnosed with a nonskin solid tumour (ICD 10 codes C00-C75, excluding C43-C44) and diagnosed during the same time interval as the cases. We excluded controls diagnosed with more than one tumour (apart from NMSC) during the study interval, and those who had a diagnosis in childhood (age<15 years).

### Data collection

Age and sex of cases and controls were captured from the registry records, as well as the date of diagnosis and site of melanoma or other solid tumour where available. Notifications of cancers were ascertained from pathology reports, medical records of National Health Service and independent hospitals and hospices and death certificates. An examination of completeness of NYCRIS skin cancer registration in 1994 demonstrated that 83% of NMSC were captured (Stefoski Mikeljevic *et al*, 2003).

NYCRIS staff routinely check all new registrations to link second (or subsequent) cancers occurring in previously registered patients. A match is made on the basis of name, date of birth and postcode of address where possible. Where there are common alternative spellings or abbreviations for a name these are also explored. If a patient has already been registered, the site and type of tumour is examined to determine if the new tumour is a new primary or represents additional information regarding a previous registration. Where the sites are different, a new registration is made. If the sites are the same, the pathological type of tumour is examined – the same type is not reregistered unless there is explicit evidence that there is a new primary. This routine linkage information was used to identify occurrence of NMSC (ICD10 C44) (specifically BCC, SCC and carcinoma *in situ* (CIS)) among all melanoma cases and other solid tumour controls.

### Statistical analysis

The primary aim of this analysis was to compare the prevalence of NMSC among patients with melanoma and other solid tumours, and to compare the magnitude of the associations according to the anatomical site of the melanoma. We therefore categorised melanoma cases into those whose melanoma occurred on the trunk, the head and neck, the upper limbs and the lower limbs and compared each group of cases to the control series. For this analysis, we were interested in the occurrence of all NMSC; hence, there was no reason *a priori* to specify whether the event of NMSC occurred before or after the event of melanoma or solid tumour diagnosis. We therefore calculated the odds ratio (OR) and 95% confidence interval (95% CI) to estimate the strength of association between period prevalence of NMSC and site of melanoma. The OR disregards any temporal relationship between the lesions. We calculated the OR using multivariable polytomous logistic regression with patients having tumours other than melanoma serving as the reference group. If more than one type of NMSC was registered for a patient (e.g. one BCC and one SCC), then we included each type in the respective NMSC type-specific analysis. If more than one NMSC of the same type was recorded, then only one of these contributed to the analysis.

In all models we adjusted for sex and age (in single years) at diagnosis of melanoma (for cases) or solid tumour (for controls), and we also included an age-squared term to adjust for residual nonlinear effects of age. All analyses were performed with SAS software (release 8.2; SAS Institute, Cary, NC, USA).

## RESULTS

We identified 349 923 control patients who had one tumour other than melanoma diagnosed between 1 January 1986 and 31 December 2000. The most commonly diagnosed tumours were those of the breast (ICD10 C45) and colon/rectum/anus (ICD10 C18-C21), each of which accounted for 14% of the registered tumours. The mean age of diagnosis was 69 years (range 15–111) and 50% of patients were male (Table 1).

There were 7084 case patients notified with cutaneous melanoma during the study period. After excluding those with incomplete notifications (missing anatomical site 491, missing histological type 2311) or ineligible histological types (LMM 279, other 229), there were 3774 patients with invasive melanomas with known site and eligible histological subtype (53% of total) for the primary analysis. Median age at melanoma diagnosis was 54 years, but was significantly higher for people with melanoma of the head and neck (69 years) compared to people with melanomas of the trunk, upper or lower limbs (53 years) (*P*<0.001, Wilcoxon test).

The overall prevalence of NMSC was 7% among melanoma cases and 3% among other solid tumour controls (crude OR 2.5, 95% CI 2.2–2.8). Adjusting for sex and the linear and nonlinear effects of age substantially increased the OR (adjusted OR 4.7, 95% CI 4.1–5.3). Among melanoma cases with NMSC, 68% were BCCs, 16% were SCCs and 16% were CIS. In contrast, among controls with keratinocyte cancers, approximately 80% were BCCs, 20% were SCCs and only one patient was diagnosed with CIS.

Associations between NMSC and melanomas at each anatomical site are presented in Table 2. On crude analysis, NMSC were more strongly associated with melanomas of the head and neck (OR 4.5) than melanomas of the trunk, arms or legs (ORs 2.1–2.5) (Table 2). After tightly adjusting for age and sex, however, patients with melanomas at all sites were approximately five-fold more likely to be diagnosed with NMSC than controls, with little variation in the magnitude of associations by anatomical site of melanoma. Similar associations were observed when analyses were restricted to specific types of NMSC (Table 2).

Further analyses of the body site distribution of NMSC and melanomas provided some evidence for anatomical colocalisation of these different types of skin cancer (Table 3). This was most apparent on the trunk, whereby patients with melanomas of the trunk were significantly more likely to have an NMSC diagnosed on the trunk (adjusted OR 12.5, 95% CI 7.2–20.2) or upper limb (adjusted OR 9.8, 95% CI 4.8–19.8) than the head and neck (adjusted OR 3.0, 95% CI 2.2–4.3). Similar patterns of colocalisation of NMSC, particularly BCCs, were observed for patients with melanomas on the upper and lower limbs. The risk of NMSC in melanoma patients at sites other than those where the melanoma arose (with the exception of the trunk and upper limb where both sites experienced a marked elevation of risk in the presence of melanoma at either site) was consistently about four-fold higher than the risk of NMSC in patients with other solid tumours.

## DISCUSSION

This study confirms that people with a diagnosis of melanoma have substantially higher risks of NMSC compared to patients with other types of cancer. This strong association was not unexpected and is similar in magnitude to risks reported in other European record linkage studies (Wassberg *et al*, 1996; Levi *et al*, 1997a; Kroumpouzos *et al*, 2000; Hemminki *et al*, 2003; Crocetti and Carli, 2004).

Our *a priori* hypothesis that patients with melanomas of the head and neck would have higher overall occurrence of NMSC than patients with melanomas of the trunk was only partially supported by these data. On crude analysis, patients with melanomas of the head and neck had substantially larger associations with NMSC than patients with melanomas at other body sites. After adjusting for linear and nonlinear effects of age, the associations between NMSC and melanoma at all body sites became markedly stronger and the apparent discrepancy between melanomas at different sites was removed.

The effects of adjusting for age were considerable; yet interpreting these effects is not straightforward. Two explanations may be considered. On the one hand, the analyses could indicate that the relationship between melanoma and NMSC was negatively confounded by age. Alternatively, if age is a necessary causal factor in the development of head and neck melanomas, as suggested by the more than 10 year age difference between patients with melanoma of the head and neck *vs* the trunk in this data set and others (Elwood and Gallagher, 1998; Bulliard, 2000), then by adjusting for age, we may actually have introduced bias to the risk estimates (Hernan *et al*, 2002). This issue cannot be resolved by further interrogation of these data.

Our new finding of colocalisation of melanoma and BCC at sites other than the head and neck has not been previously reported. To our knowledge, only one other study has investigated this possibility, although it was hampered by a short duration of follow-up and a small sample size calling the observed null result into question (Kroumpouzos *et al*, 2000). Assuming that our finding was not the result of chance, the question arises as to why melanomas and BCC should tend to arise on the same anatomical sites. One possibility is heightened awareness or surveillance of the body site where the first tumour was excised. This possibility is supported by the frequent diagnosis of *in situ* keratinocyte carcinomas among melanoma patients compared with their virtual absence in controls. Moreover, a recent Australian study has shown a marked increase in the incidence of BCC on less sun exposed body sites when active skin screening is undertaken (Valery *et al*, 2004).

Although heightened skin cancer surveillance among melanoma patients might partially explain colocalisation, this phenomenon may also reflect common variation in the component causes of both melanoma and BCC arising at different anatomical sites. As with melanoma, the association between sun exposure and BCC is complex and is likely to be modified by genetic factors and the anatomical location of the target cell (Lear *et al*, 1997a, 1997b; Ramachandran *et al*, 2001a, 2001b). For both melanoma and BCC, those tumours occurring on the head and neck are more likely to overexpress p53 protein then their truncal counterparts (D'Errico *et al*, 1997; Whiteman *et al*, 1998). Similarly, both melanomas and BCC arise at considerably older ages on the head and neck than on the trunk (Ramachandran *et al*, 2001a). These various observations support the view that melanomas and BCCs occurring on the trunk share similar causes, which appear to differ from those required to induce BCCs and melanomas of the head and neck.

We considered the possibility that our findings may be influenced by other sources of error. Under-registration of NMSC is a potential problem, although recent analyses of the NYCRIS suggest that more than 80% of NMSC are captured in this population (Stefoski Mikeljevic *et al*, 2003). Increased surveillance of melanoma patients may increase the likelihood of diagnosing NMSC compared with control patients, but there is no reason to suppose that registration is differentially related to the site of the melanoma.

Another potential source of error stems from the fact that almost half of the melanoma notifications to the registry were excluded from these analyses, mostly due to missing information about the histological type of melanoma that was considered important *a priori*. We tested the effect of this restriction by reanalysing the data after including all cases with missing histological information. We found no alteration in the pattern of results to those reported in the tables.

In conclusion, these data provide support for the notion that melanomas and BCCs share similar causal factors, but suggest that the associations between different types of skin cancer differ according to the anatomical site of the lesion and the age of the host.

## Figures and Tables

**Table 1:** Descriptive statistics for melanoma cases and solid tumour controls

**Cases**	**All melanomas^a^**	**Eligible melanomas^b^**
*Head and neck*		
*N* (%) of all melanomas	1193 (19)	512 (14)
*N* (%) female	632 (53)	268 (52)
Mean (median) age at diagnosis	67 (71)	64 (69)
*N* (%) with keratinocyte cancers	146 (12)	64 (13)

*Trunk*		
*N* (%) of all melanomas	1550 (24)	1018 (27)
*N* (%) female	593 (38)	386 (38)
Mean (median) age at diagnosis	52 (53)	52 (53)
*N* (%) with keratinocyte cancers	97 (6)	65 (6)

*Upper limbs*		
*N* (%) of all melanomas	1189 (19)	749 (20)
*N* (%) female	765 (64)	487 (65)
Mean (median) age at diagnosis	54 (54)	53 (52)
*N* (%) with keratinocyte cancers	79 (7)	55 (7)

*Lower limbs*		
*N* (%) of all melanomas	2432 (38)	1495 (40)
*N* (%) female	1944 (80)	1205 (81)
Mean (median) age at diagnosis	55 (55)	53 (53)
*N* (%) with keratinocyte cancers	142 (6)	92 (6)

**Controls**		
*Solid tumours*		
*N*	349 923	
*N* (%) female	173 795 (50)^c^	
Mean (median) age at diagnosis	69 (70)	
*N* (%) with keratinocyte cancers	10 754 (3)	

a Excluding those for whom site was unknown.

b Site known, eligible histological type (not LMM, acral lentiginous, desmoplastic or other ineligible types).

c Sex missing for 151 patients.

**Table 2:** Odds ratios (and 95% CI) of keratinocyte cancer among cases with melanoma of the head/neck, trunk, upper and lower limbs compared to controls with other solid tumours

	**Controls (*N*=349 923)**	**Head and neck melanoma cases (*N*=512)**			**Trunk melanoma cases (*N*=1018)**			**Upper limb melanoma cases (*N*=749)**			**Lower limb melanoma cases (*N*=1569)**		
	**%**	**%**	**OR (95% CI)^a^**	**Adj OR (95% CI)^b^**	**%**	**OR (95% CI)^a^**	**Adj OR (95% CI)^b^**	**%**	**OR (95% CI)^a^**	**Adj OR (95% CI)^b^**	**%**	**OR (95% CI)^a^**	**Adj OR (95% CI)^b^**
Any keratinocyte cancers	3.07	12.50	4.51 (3.47–5.86)	5.26 (4.01–6.89)	6.39	2.15 (1.67–2.77)	4.53 (3.50–5.87)	7.34	2.50 (1.90–3.29)	5.21 (3.93–6.92)	6.15	2.07 (1.67–2.56)	4.26 (3.43–5.30)
SCC^c^	0.60	2.54	4.68 (2.69–8.13)	5.33 (3.04–9.33)	1.18	2.03 (1.15–3.59)	4.85 (2.72–8.64)	1.07	1.86 (0.92–3.74)	4.79 (2.37–9.70)	0.54	0.92 (0.46–1.84)	2.49 (1.24–5.02)
BCC^d^	2.55	10.16	4.40 (3.30–5.87)	5.15 (3.84–6.92)	5.80	2.35 (1.80–3.06)	4.82 (3.68–6.32)	5.21	2.13 (1.54–2.95)	4.31 (3.09–5.99)	4.68	1.89 (1.49–2.41)	3.74 (2.92–4.78)

a Odds ratio and 95% confidence interval. The reference group was people with no keratinocyte cancers in each analysis.

b Odds ratio and 95% confidence interval adjusted for exact age (in years), age squared and sex.

c In total, 9080 people were excluded from the SCC analysis because they had no SCC diagnosis but a diagnosis of another keratinocyte cancers.

d In total, 1975 people were excluded from the BCC analysis because they had a diagnosis of a keratinocyte cancer other than BCC.

**Table 3:** Odds ratio (and 95% CI) of keratinocyte cancers occurring at specific anatomic sites among cases with melanoma of the head/neck, trunk, upper and lower limbs compared to controls with other solid tumours

	**Controls (*N*=349 923)**	**Head and neck melanoma cases (*N*=512)**			**Trunk melanoma cases (*N*=1018)**			**Upper limb melanoma cases (*N*=749)**			**Lower limb melanoma cases (*N*=1569)**		
**Keratinocytic cancer site**	**%**	**%**	**OR (95% CI)^a^**	**Adj OR (95% CI)^b^**	**%**	**OR (95% CI)^a^**	**Adj OR (95% CI)^b^**	**%**	**OR (95% CI)^a^**	**Adj OR (95% CI)^b^**	**%**	**OR (95% CI)^a^**	**Adj OR (95% CI)**
*Head and neck*													
All keratinocyte cancers	2.46	10.35	4.58 (3.44–6.09)	5.33 (3.97–7.14)	3.44	1.41 (1.01–1.98)	3.03 (2.15–4.27)	4.14	1.71 (1.19–2.45)	3.68 (2.55–5.31)	2.74	1.12 (0.82–1.53)	2.40 (1.75–3.29)
BCC only^d^	2.15	9.49	4.77 (3.53–6.44)	5.58 (4.10–7.60)	3.44	1.62 (1.15–2.28)	3.47 (2.45–4.92)	3.34	1.57 (1.05–2.36)	3.36 (2.22–5.08)	2.50	1.17 (0.84–1.62)	2.44 (1.74–3.41)
SCC only^c^	0.39	1.97	5.16 (2.66–10.01)	5.89 (3.02–11.51)	0.63	1.62 (0.72–3.62)	3.89 (1.73–8.74)	0.72	1.85 (0.77–4.47)	5.23 (2.15–12.71)	0.28	0.73 (0.27–1.96)	2.32 (0.87–6.24)

*Trunk*													
All keratinocyte cancers	0.29	0.78	4.03 (1.50–10.82)	4.44 (1.66–11.93)	1.77	9.22 (5.75–14.78)	12.48 (7.15–20.19)	1.34	6.93 (3.70–12.99)	9.87 (5.23–18.61)	0.80	4.14 (2.37–7.35)	6.04 (3.38–10.81)
BCC only^d^	0.17	0.88	5.16 (1.92–13.85)	5.81 (2.16–15.62)	1.55	9.90 (5.43–15.24)	12.32 (7.28–20.85)	1.14	6.66 (3.30–13.43)	9.42 (4.64–19.13)	0.78	4.53 (2.49–8.24)	6.48 (3.53–11.90)
SCC only^c^	0.03	0.00	N/C	N/C	0.21	7.49 (1.84–30.44)	11.94 (2.86–49.92)	0.14	5.14 (0.72–36.95)	9.83 (1.35–71.58)	0.00	N/C	N/C

*Upper limbs*													
All keratinocyte cancers	0.15	0.59	3.95 (1.27–12.27)	4.39 (1.40–13.71)	0.79	5.28 (2.62–10.64)	9.77 (4.81–19.84)	1.07	7.19 (3.56–14.50)	13.76 (6.76–28.00)	0.27	1.79 (0.67–4.79)	3.56 (1.32–9.59)
BCC only^d^	0.07	0.00	N/C	N/C	0.63	9.66 (4.28–21.79)	15.76 (6.90–36.02)	0.43	6.63 (2.12–20.78)	10.62 (3.35–33.67)	0.14	2.19 (0.54–8.81)	3.45 (0.85–14.08)
SCC only^c^	0.09	0.44	5.06 (1.26–20.30)	5.90 (1.46–23.84)	0.10	1.18 (0.17–8.43)	2.67 (0.37–19.11)	0.14	1.62 (0.23–11.58)	4.07 (0.57–29.09)	0.07	0.80 (0.11–5.72)	2.16 (0.30–15.40)

*Lower limbs*													
All keratinocyte cancers	0.17	0.39	2.27 (0.56–9.11)	2.49 (0.62–10.03)	0.29	1.71 (0.55–5.32)	3.85 (1.23–12.02)	0.40	2.33 (0.75–7.25)	3.85 (1.25–12.02)	1.74	10.24 (6.89–15.21)	14.39 (9.59–21.58)
BCC only^d^	0.10	0.22	2.14 (0.30–15.30)	2.47 (0.35–17.66)	0.31	3.03 (0.97–9.44)	6.26 (1.99–19.71)	0.14	1.38 (0.19–9.87)	2.22 (0.31–15.90)	0.92	8.90 (5.11–15.52)	12.07 (6.85–21.25)
SCC only^c^	0.07	0.00	N/C	N/C	0.00	N/C	N/C	0.00	N/C	N/C	0.14	1.98 (0.49–7.88)	3.27 (0.81–13.25)

a Odds ratio and 95% confidence interval. The reference group was people with no keratinocyte cancers in each analysis.

b Odds ratio and 95% confidence interval adjusted for exact age (in years), age squared and sex.

c In total, 9080 people were excluded from the SCC analysis because they had no SCC diagnosis but a diagnosis of another keratinocyte cancers.

d In total, 1975 people were excluded from the BCC analysis because they had a diagnosis of a keratinocyte cancer other than BCC. N/C=not calculated due to a zero cell in the control group.
